# Supplementary material for: Spatial Methods for Inferring Extremes in Dengue Outbreak Risk in Singapore
Source: Viruses. 2022 Nov 4;14(11):2450. doi: 10.3390/v14112450 (PMC9695662; doi:10.3390/v14112450)
Supplement: Supplementary file 1 [file viruses-14-02450-s001.zip › viruses-1922961-supplementary.pdf]

Supplementary Information to: Spatial methods to infer extremes in dengue outbreak risk in Singapore

1. Calculation of absolute and relative humidity

$$\text{AH} = \frac{1000(6.11)(10^{T_1})(100)}{461.5(T_c + 273.16)}$$

where T_c denotes the daily mean temperature, and

$$T_1 = \frac{7.5(T_d)}{237.7 + T_d}$$

where T_d denotes the dew point temperature and can be estimated using the equation

$$T_d = \frac{237.7\ln(E) - 430.22}{19.08 - \ln(E)}, \text{ where } E = \frac{RH(E_s)}{100},\ E_s = 6.11(10^{T_2}) \text{ and } T_2 = \frac{7.5T_c}{237.7 + T_c}$$

2. Trend surfaces and pairwise deviance of other max-stable models

Table S1: Pairwise deviance from the other max-stable models.

Model[1]	Trend surfaces[2]	Relative Deviance[3]
Smith (M5)	$\mu(x) = \beta_{\mu,0} + \beta_{\mu,1}X + \sum_{i=2}^{q} \beta_{\mu,i} Q_i(x)$ $\sigma^*(x) = \beta_{\sigma^*,0} + \beta_{\sigma^*,1}X + \beta_{\sigma^*,2}Y + \sum_{i=3}^{q} \beta_{\mu,i} Q_i(x)$	1.465
Smith (M6)	$\mu(x) = \beta_{\mu,0} + \beta_{\mu,1}X + \sum_{i=2}^{q} \beta_{\mu,i} Q_i(x)$ $\sigma^*(x) = \beta_{\sigma^*,0} + \beta_{\sigma^*,1}X + \beta_{\sigma^*,2}Y + \beta_{\sigma^*,3}X * Y + \sum_{i=4}^{q} \beta_{\mu,i} Q_i(x)$	1.582
Schlather (M7)	$\mu(x) = \beta_{\mu,0} + \beta_{\mu,1}X + \sum_{i=2}^{q} \beta_{\mu,i} Q_i(x)$ $\sigma^*(x) = \beta_{\sigma^*,0} + \beta_{\sigma^*,1}X + \sum_{i=2}^{q} \beta_{\mu,i} Q_i(x)$	2.484
Smith (M8)	$\mu(x) = \beta_{\mu,0} + \beta_{\mu,1}X + \sum_{i=2}^{q} \beta_{\mu,i} Q_i(x)$ $\sigma^*(x) = \beta_{\sigma^*,0} + \beta_{\sigma^*,1}X + \sum_{i=2}^{q} \beta_{\mu,i} Q_i(x)$	2.719
Schlather (M9)	$\mu(x) = \beta_{\mu,0} + \beta_{\mu,1}X + \beta_{\mu,2}Y + \beta_{\mu,3}X * Y + \sum_{i=4}^{q} \beta_{\mu,i} Q_i(x)$ $\sigma^*(x) = \beta_{\sigma^*,0} + \beta_{\sigma^*,1}X + \sum_{i=2}^{q} \beta_{\mu,i} Q_i(x)$	2.386
Brown-Resnick (M10)	$\mu(x) = \beta_{\mu,0} + \beta_{\mu,1}X + \beta_{\mu,2}Y + \beta_{\mu,3}X * Y + \sum_{i=4}^{q} \beta_{\mu,i} Q_i(x)$ $\sigma^*(x) = \beta_{\sigma^*,0} + \beta_{\sigma^*,1}X + \sum_{i=2}^{q} \beta_{\mu,i} Q_i(x)$	2.466

Smith (M11)	$\mu(x) = \beta_{\mu,0} + \beta_{\mu,1}X + \beta_{\mu,2}Y + \beta_{\mu,3}X * Y + \sum_{i=4}^{q} \beta_{\mu,i}\, Q_i(x)$<br>$\sigma^*(x) = \beta_{\sigma^*,0} + \beta_{\sigma^*,1}X + \sum_{i=2}^{q} \beta_{\mu,i}\, Q_i(x)$	2.545
Schlather (M12)	$\mu(x) = \beta_{\mu,0} + \beta_{\mu,1}X + \sum_{i=2}^{q} \beta_{\mu,i}\, Q_i(x)$<br>$\sigma^*(x) = \beta_{\sigma^*,0} + \beta_{\sigma^*,1}Y + \sum_{i=2}^{q} \beta_{\mu,i}\, Q_i(x)$	3.229
Brown-Resnick (M13)	$\mu(x) = \beta_{\mu,0} + \beta_{\mu,1}X + \sum_{i=2}^{q} \beta_{\mu,i}\, Q_i(x)$<br>$\sigma^*(x) = \beta_{\sigma^*,0} + \beta_{\sigma^*,1}Y + \sum_{i=2}^{q} \beta_{\mu,i}\, Q_i(x)$	2.043
Smith (M14)	$\mu(x) = \beta_{\mu,0} + \beta_{\mu,1}X + \sum_{i=2}^{q} \beta_{\mu,i}\, Q_i(x)$<br>$\sigma^*(x) = \beta_{\sigma^*,0} + \beta_{\sigma^*,1}Y + \sum_{i=2}^{q} \beta_{\mu,i}\, Q_i(x)$	3.847
Brown-Resnick (M15)	$\mu(x) = \beta_{\mu,0} + \beta_{\mu,1}Y + \sum_{i=2}^{q} \beta_{\mu,i}\, Q_i(x)$<br>$\sigma^*(x) = \beta_{\sigma^*,0} + \beta_{\sigma^*,1}Y + \sum_{i=2}^{q} \beta_{\mu,i}\, Q_i(x)$	1.475
Smith (M16)	$\mu(x) = \beta_{\mu,0} + \beta_{\mu,1}Y + \sum_{i=2}^{q} \beta_{\mu,i}\, Q_i(x)$<br>$\sigma^*(x) = \beta_{\sigma^*,0} + \beta_{\sigma^*,1}Y + \sum_{i=2}^{q} \beta_{\mu,i}\, Q_i(x)$	1.475
Smith (M17)	$\mu(x) = \beta_{\mu,0} + \beta_{\mu,1}Y + \sum_{i=2}^{q} \beta_{\mu,i}\, Q_i(x)$<br>$\sigma^*(x) = \beta_{\sigma^*,0} + \beta_{\sigma^*,1}X + \sum_{i=2}^{q} \beta_{\mu,i}\, Q_i(x)$	2.391
Schlather (M18)	$\mu(x) = \beta_{\mu,0} + \beta_{\mu,1}Y + \beta_{\mu,2}Y + \sum_{i=3}^{q} \beta_{\mu,i}\, Q_i(x)$<br>$\sigma^*(x) = \beta_{\sigma^*,0} + \beta_{\sigma^*,1}X + \sum_{i=2}^{q} \beta_{\mu,i}\, Q_i(x)$	1.433
Smith (M19)	$\mu(x) = \beta_{\mu,0} + \beta_{\mu,1}Y + \beta_{\mu,2}Y + \sum_{i=3}^{q} \beta_{\mu,i}\, Q_i(x)$<br>$\sigma^*(x) = \beta_{\sigma^*,0} + \beta_{\sigma^*,1}X + \sum_{i=2}^{q} \beta_{\mu,i}\, Q_i(x)$	1.456
Smith (M20)	$\mu(x) = \beta_{\mu,0} + \beta_{\mu,1}Y + \beta_{\mu,2}Y + \sum_{i=3}^{q} \beta_{\mu,i}\, Q_i(x)$<br>$\sigma^*(x) = \beta_{\sigma^*,0} + \beta_{\sigma^*,1}X + \beta_{\sigma^*,2}Y + \sum_{i=3}^{q} \beta_{\mu,i}\, Q_i(x)$	1.505

[1]Estimated max-stable models under the pairwise likelihood approach.
[2]Trend surfaces denote the pointwise distribution imposed on each spatial unit where dengue case counts were collected. X, Y denotes the horizontal and vertical coordinates respectively and Q refers to the spatial covariates as described in Section 3.1. No restrictions were imposed on the scale parameter ξ.
[3]The relative deviance is given by the ratio of the pairwise deviance for the model of interest over the best model (M1).

3. Marginal distributions appropriately modelled under spatial dependence structure

We assessed the marginal distributions via two functions: (i) the quantile-quantile (QQ) plot and (ii) the extremal coefficient estimates plot from the F-madogram. We generated QQ plots for all hexagons in the model, nine of which are randomly selected and shown in Figure S1. The plots indicate some degree of overestimation, which is likely due to the limited data points in each location. In addition, we estimated empirically the pairwise extremal coefficient between each pair of sites from the binned F-madogram (Figure S2). The plot demonstrates spatial dependence between dengue case maxima at all distances.

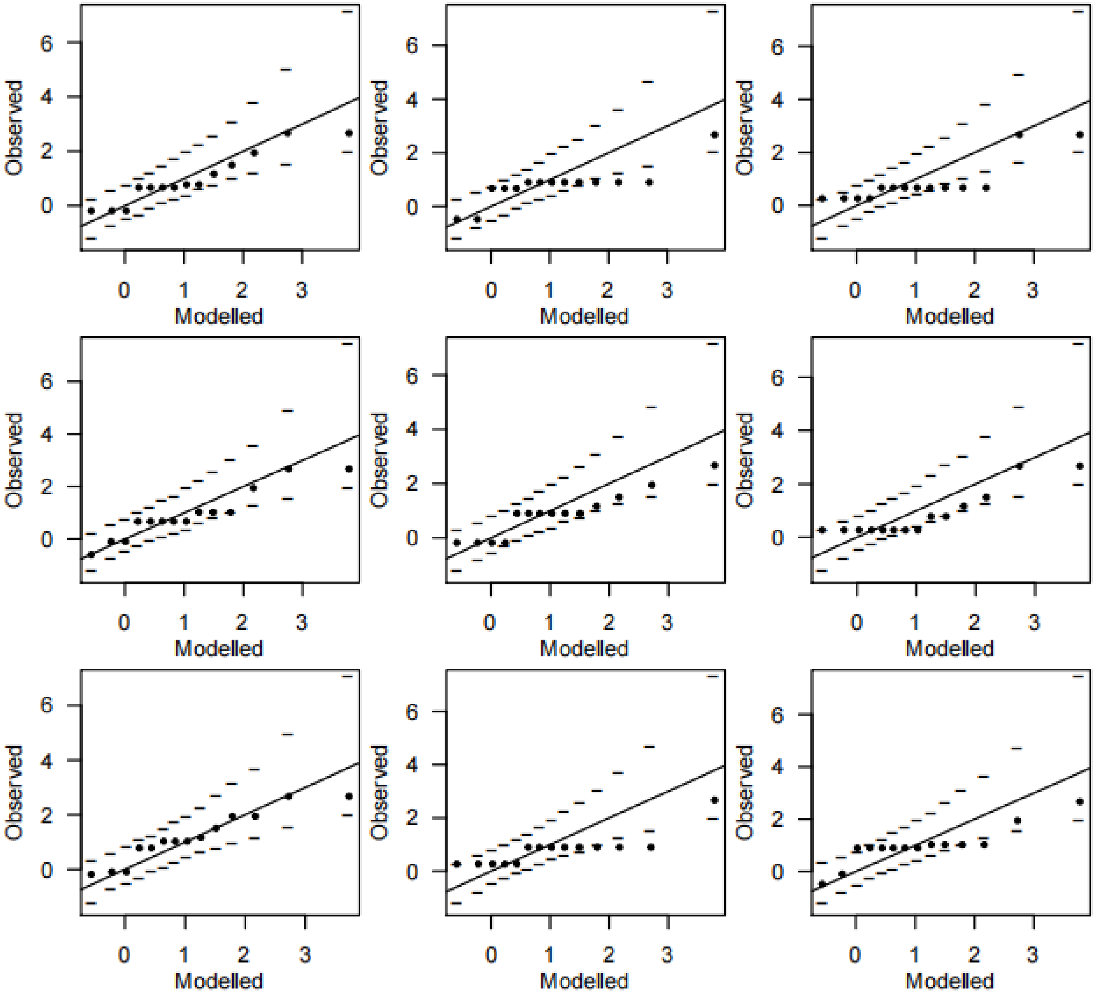

Figure S1. QQ plot for 9 random locations. The dots represent the observed pairwise annual weekly maximum dengue case counts in each year compared against the simulated fitted model. The dotted lines represent the 95% confidence interval of the simulated fitted model. The solid line represents the line of equality.

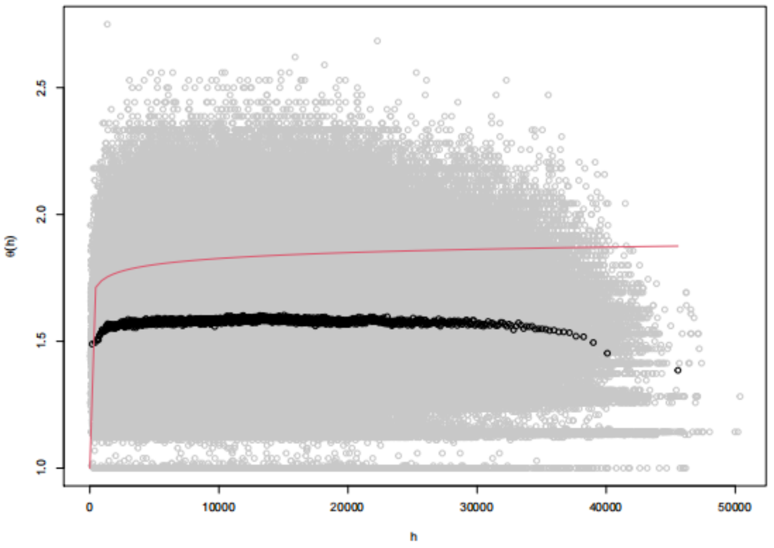

Figure S2: Extremal coefficient with unit Gumbel margins from the best model (**M1**). The red line represents the theoretical extremal coefficient, the gray points represent the pairwise estimates and the black solid circles represent the binned estimates.

4. Plot of univariate GEV values in each spatial unit

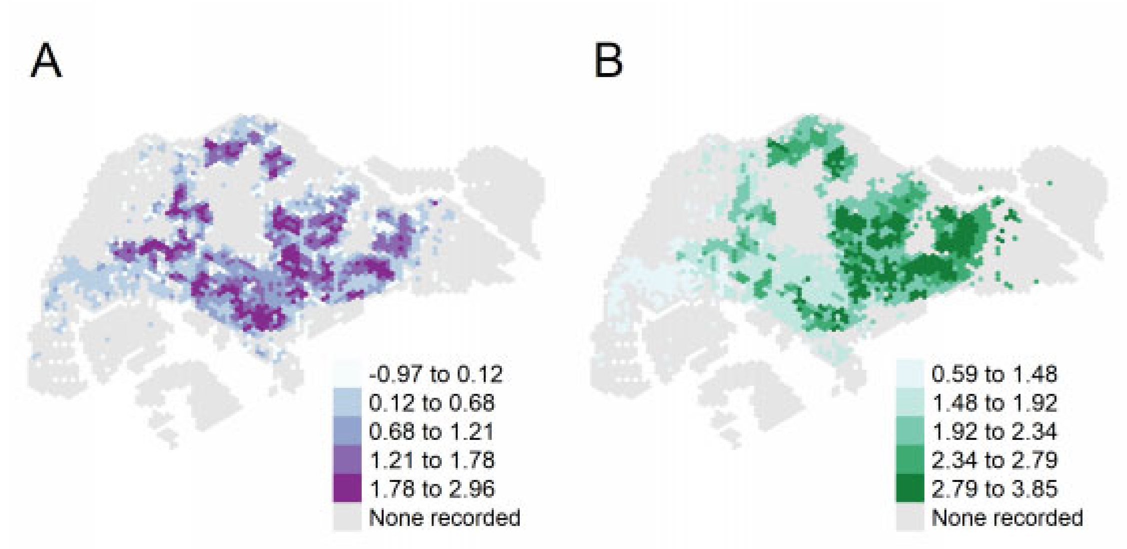

Figure S3: (A) Location and **(B)** Scale parameters for each spatial location derived from the generalized extreme value distribution under the best model specification (M1).
